# YAP1 Recognizes Inflammatory and Mechanical Cues to Exacerbate Benign Prostatic Hyperplasia via Promoting Cell Survival and Fibrosis

**DOI:** 10.1002/advs.202304274

**Published:** 2023-12-04

**Authors:** Dongxu Lin, Changcheng Luo, Pengyu Wei, An Zhang, Mengyang Zhang, Xiaoliang Wu, Bolang Deng, Zhipeng Li, Kai Cui, Zhong Chen

**Affiliations:** ^1^ Department and Institute of Urology Tongji Hospital Tongji Medical College Huazhong University of Science and Technology Wuhan 430030 China; ^2^ Department of Geriatrics Tongji Hospital Tongji Medical College Huazhong University of Science and Technology Wuhan 430030 China; ^3^ Department of Rehabilitation Tongji Hospital Tongji Medical College Huazhong University of Science and Technology Wuhan 430030 China

**Keywords:** benign prostatic hyperplasia, cytoskeleton remodeling, fibrosis, inflammation, yes‐associated protein 1

## Abstract

Chronic prostatic inflammation promotes cell survival and fibrosis, leading to benign prostatic hyperplasia (BPH) with aggravated urinary symptoms. It is investigated whether yes‐associated protein 1 (YAP1), an organ size controller and mechanical transductor, is implicated in inflammation‐induced BPH. The correlation between YAP1 expression and fibrosis in human and rat BPH specimens is analyzed. Furthermore, the effects of YAP1 activation on prostatic cell survival and fibrosis, as well as the underlying mechanism, are also studied. As a result, total and nuclear YAP1 expression, along with downstream genes are significantly upregulated in inflammation‐associated human and rat specimens. There is a significant positive correlation between YAP1 expression and the severity of fibrosis or clinical performance. YAP1 silencing suppresses cell survival by decreasing cell proliferation and increasing apoptosis, and alleviates fibrosis by reversing epithelial‐mesenchymal transition (EMT) and extracellular matrix (ECM) deposition in prostatic BPH‐1 and WPMY‐1 cells. Mechanistically, inflammatory stimulus and rigid matrix stiffness synergistically activate the RhoA/ROCK1 pathway to provoke cytoskeleton remodeling, thereby promoting YAP1 activation to exacerbate BPH development. Overall, inflammation‐triggered mechanical stiffness reinforcement activates the RhoA/ROCK1/F‐actin/YAP1 axis, thereby promoting prostatic cell survival and fibrosis to accelerate BPH progression.

## Graphical abstract

Chronic inflammation promotes activation of the RhoA/ROCK1 pathway and facilitates YAP1 nuclear translocation to interact with TEAD1, thereby promoting cell survival and fibrosis. The fibrotic microenvironment reinforces mechanical stiffness and, in combination with inflammatory stimulus, synergistically promotes cytoskeleton‐dependent YAP1 reactivation. Targeting YAP1 may represent a promising approach to break the vicious cycle and alleviate inflammation‐associated benign prostatic hyperplasia (BPH) progression.

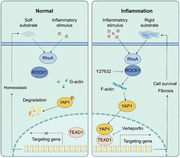

## 1 Introduction

Benign prostatic hyperplasia (BPH) is an age‐dependent disorder with a prevalence of 50% in men over fifty years old, and the prevalence increases to as high as 80% in men over eighty years old.^[^ ^1^ ^]^ It is typically featured as a histological enlargement of primarily transitional zone of prostate due to uncontrolled proliferation of epithelial and stromal cells, which may be responsible for the occurrence of bladder outlet obstruction and consequent lower urinary tract symptoms (LUTS).^[^ ^2^ ^]^ So far, there is still no consensus regarding the cellular and molecular mechanisms underlying the pathogenesis of BPH. Estrogen/testosterone imbalance, mesenchymal stem cell reawakensing, and tissue remodeling may partially explain the occurrence and progression of BPH,^[^ ^2^, ^3^ ^]^ however, many pathological processes remain unclear.

Recently, the role of inflammation in BPH has attracted broad attention. Prostatic inflammation is considered to increase BPH progressive risk, mainfesting as exacerbated LUTS performance and an increased risk of acute urine retention.^[^ ^4^ ^]^ Chronic inflammation has been proven to drive prostatic tissue hyperplasia and fibrosis, characterized by reactive stroma and extracellular matrix (ECM) deposition.^[^ ^5^ ^]^ Epithelial‐mesenchymal transition (EMT) is a process in which cells of epithelial origin losing their cell‐cell adhesion features and acquiring a mesenchymal phenotype, which is characterized by increased motility and ECM production.^[^ ^6^ ^]^ Phenotype transition, including EMT and fibroblast‐myofibroblast transition (FMT), along with the accumulation of ECM proteins by activated myofibroblasts, exacerbate fibrotic damage and stiffen the tissue. The ECM provides structural support to tissues and helps regulate cellular behavior.^[^ ^7^ ^]^ Inflammation‐induced fibrosis progression, which involves processes such as EMT and FMT, contributes to ECM deposition in the periurethral prostate. Subsequently, this reinforces matrix stiffness, reduces urethral compliance, and eventually causes LUTS.^[^ ^8^ ^]^ However, the detailed mechanism by which inflammation mediates prostatic fibrosis is not well understood.

Ras homolog gene family member A (RhoA) and its downstream effector Rho‐associated coiled‐coil containing kinase 1 (ROCK1) are members of the Rho family of small GTPases, which control actin cytoskeleton dynamics, and affect cell shape and various cellular behaviors.^[^ ^9^ ^]^ Blocking the RhoA/ROCK1 pathway can alleviate BPH progression.^[^ ^10^ ^]^ Interestingly, RhoA/ROCK1‐associated actin cytoskeleton remodeling activates YAP1 protein, the core effector of Hippo pathway.^[^ ^11^ ^]^ The latter is an evolutionally conserved pathway that is implicated in many crucial biological processes, such as organ size control, cellular homeostasis, and tissue regeneration.^[^ ^12^ ^]^ YAP1 is affected by multiple upstream signals, including mechanical signals, cell adhesion and inflammatory molecules. After that, dephosphorylated YAP1 is translocated into the cell nucleus, where it interacts with various transcription factors, such as TEA domain DNA‐binding family members (TEADs), to regulate the expression of genes related to cell survival, differentiation, tissue remodeling and fibrosis.^[^ ^12b^ ^]^ Hyperactivation of YAP1 promotes cell survival by inhibiting apoptosis, which is partially attributed to the transcriptional activation of anti‐apoptotic factors in the Bcl‐2 family member.^[^ ^13^ ^]^ In addition, YAP activation triggers fibrogenesis by promoting myofibroblast differentiation and ECM deposition.^[^ ^14^ ^]^

Despite emerging evidence implying inflammation or ECM stiffness‐mediated crosstalk between YAP1 activation and fibrogenesis,^[^ ^15^ ^]^ questions that remain unclear include whether YAP1 activation affects BPH progression through regulating actin cytoskeleton rearrangement. Hence, the current study aims to investigate the role of YAP1 in prostatic cell survival and fibrosis in the context of inflammatory and mechanical stimuli, so as to provide novel insights into inflammation‐associated BPH progression.

## 2 Results

### 2.1 Elevated YAP Expression Correlates with ECM Remodeling and LUTS Performance in Patients with Inflamed‐BPH

**Figure 1:**
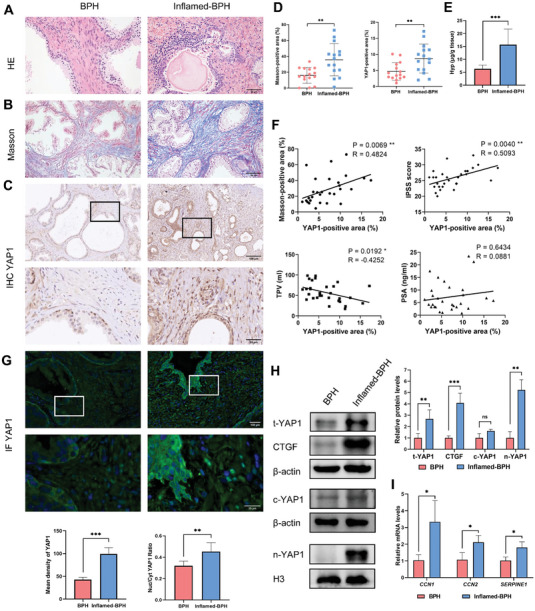
Elevated YAP expression correlates with tissue fibrosis and LUTS performance in inflamed‐BPH patients. A) Representative images of HE‐stained prostate tissue specimens from the BPH and inflamed‐BPH groups. B) Representative images of Masson‐stained tissues showing the collagen fiber deposition. C) Representative images showing in‐situ YAP1 expression in the prostate tissues. D) Statistical analysis to compare the difference of Masson/YAP1‐positive area between the indicated groups. E) Comparison of Hyp contents between two groups. F) Correlation analysis of the YAP1‐positive area with the Masson‐positive area, IPSS score, TPV, and PSA level. G) IF analysis to investigate the expression and cellular location of YAP1 by determining average intensity and nucleus/cytoplasm (Nuc/Cyt) ratio of YAP1. H) Immunoblotting analysis to investigate the levels of total, cytoplasmic, and nuclear YAP1 proteins (t‐YAP1, c‐YAP1, n‐YAP1, respectively), and downstream CTGF protein. I) RT‐PCR analysis of the expression of YAP1 downstream genes (*CCN1*, *CCN2*, *SERPINE1*). Data were presented as mean ± SD of 4 to 9 independent experiments. Two‐tailed Student's *t*‐test was used for D,E,G,H,I). Pearson correlation analysis was used for F). *p < 0.05, ** *p* < 0.01, *** *p* < 0.001.

The BPH patients were categorized into the non‐inflamed BPH (BPH) group and BPH with inflammation (inflamed‐BPH) group based on the histological features of the prostate. As a result, prostate specimens from the inflamed‐BPH groups showed massive infiltration of inflammatory cells, increased collagen fiber deposition, and enhanced expression of YAP1 compared to those from the control BPH group (**Figure** **1**A–D). Additionally, the inflamed‐BPH specimens exhibited a notable increase in the concentration of hydroxyproline (Hyp), a considerable indicator of tissue collagen content (Figure 1E). Correlation analysis uncovered that YAP1‐positive area ratio was positively correlated with Masson‐positive collagen fiber area ratio and international prostate symptom score (IPSS), and negatively correlated with total prostate volume (TPV). No statistical correlation was observed between YAP1 expression and prostate specific antigen (PSA) level (Figure 1F).

Immunofluorescence (IF) analysis revealed a substantial increase in both the average fluorescence intensity and the nucleus/cytoplasm (Nuc/Cyt) ratio of YAP1 in the inflamed‐BPH group, surpassing those observed in the BPH group (Figure 1G). Immunoblotting analysis further confirmed that the tissue homogenates of the inflamed‐BPH groups displayed elevated levels of total and nuclear YAP1 proteins, as well as the downstream protein, connective tissue growth factor (CTGF), which is known for its pro‐fibrotic and ECM remodeling effects (Figure 1H). Furthermore, real‐time quantitative PCR (RT‐PCR) results confirmed a significant upregulation in the mRNA expression of well‐known YAP1 downstream genes, including *CCN1*, *CCN2*, and *SERPINE1*, in the specimens from the inflamed‐BPH group (Figure 1I). These findings emphasize the involvement of YAP1 activation in the pathogenesis of inflammation‐associated BPH, which may be attributed to the alteration in collagen remodeling.

### 2.2 Inflammation Causes Stromal Fibrosis and YAP1 Nuclear Translocation in Rat Prostate Tissues

**Figure 2:**
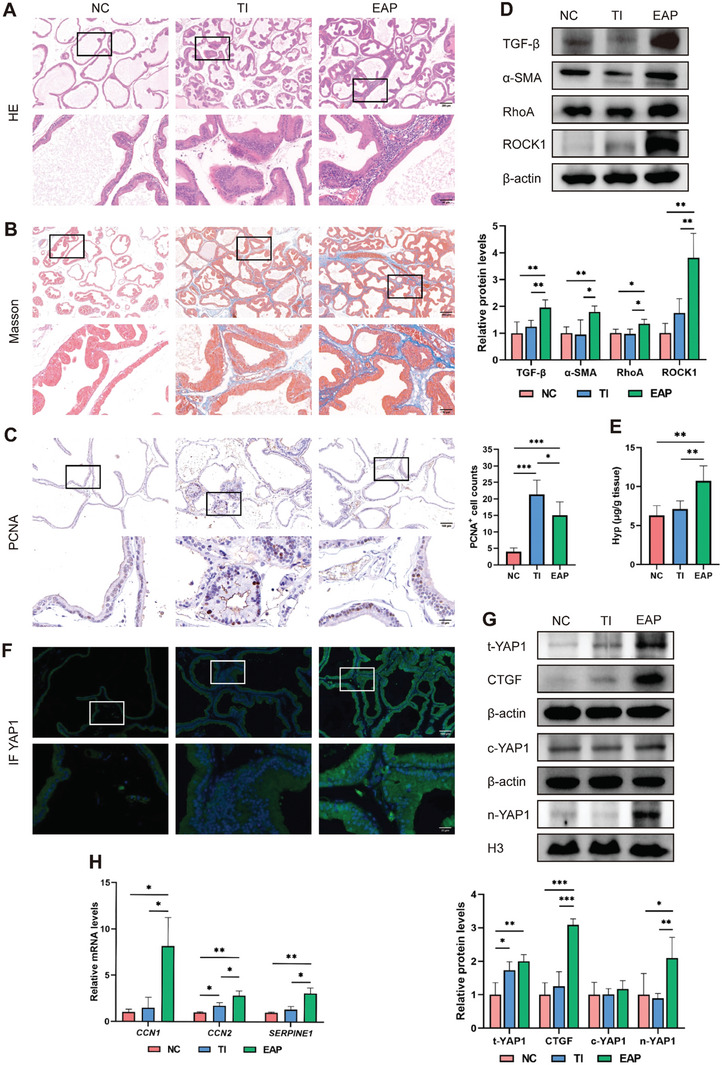
Inflammation causes stromal fibrosis and YAP1 nuclear translocation in rat prostate tissues. A) Representative images of HE staining observing the histological features in the rat prostate tissues of negative control (NC), testosterone induction (TI), and experimental autoimmune prostatitis (EAP) groups. B) Representative images of Masson staining showing the collagen fiber deposition. C) IHC analysis to identify PCNA‐expressed proliferating cells, and the positive cells were counted. D) Immunoblotting analysis to compare the expression levels of fibrosis‐associated proteins (TGF‐β/α‐SMA) and RhoA/ROCK1 pathway among the indicated groups. E) Comparison of Hyp contents among three groups. F) Representative images showing the expression and sublocation of YAP1. G) Immunoblotting analysis of the levels of total, cytoplasmic, and nuclear YAP1 proteins and downstream CTGF protein. H) RT‐PCR analysis of the mRNA expression of YAP1 downstream genes (*CCN1*, *CCN2*, *SERPINE1*). Data were presented as mean ± SD of 3 to 6 independent experiments. Two‐tailed Student's *t*‐test was used for C, D, E, G, H). *p < 0.05, ** *p* < 0.01, *** *p* < 0.001.

Different induction methods caused distinct pathological features of rat prostate samples. For example, the prostates of testosterone induction (TI) groups exhibited prominent proliferation of glandular epithelial cells, whereas the prostates of experimental autoimmune prostatitis (EAP) groups performed diffuse inflammatory cells infiltration and microvascular congestion (**Figure** **2**A). The collagen fiber was obviously more deposited in the prostate stroma of the EAP model compared to that in the negative control (NC) and TI models (Figure 2B). The Histoscore protocol was employed to assess histopathological damages in the prostate samples. As a result, the Histoscores were significantly higher in the TI (52.29 ± 6.80) and EAP (40.13 ± 8.14) groups compared with the NC group (23.58 ± 9.15), indicating considerable histological damage due to respective stimuli (Table S1, Supporting Information). Notably, the TI group exhibited severe epithelial lesions, while the EAP group displayed both epithelial and stromal lesions concurrently. The number of PCNA‐expressed proliferating cells in the TI and EAP groups was significantly higher than that in the NC group (Figure 2C). Induction of EAP also upregulated the pro‐fibrotic factor TGF‐β and the myofibroblast marker α‐SMA, activated the RhoA/ROCK1 pathway (Figure 2D), and significantly increased the levels of collagen marker Hyp compared with the NC and TI groups (Figure 2E).

The expression pattern of YAP1 among different BPH model were further analyzed. The prostate samples from EAP groups performed enhanced YAP1 expression and its nuclear translocation, subsequently leading to the transcriptional activation of its downstream target CTGF (Figure 2F,G). Moreover, the mRNA levels of YAP1 downstream genes (*CCN1*, *CCN2*, and *SERPINE1*), well known for their tissue remodeling function, were significantly increased in the EAP groups (Figure 2H). These notable findings signify active cell proliferation, myofibroblast phenotype conversion and matrix remodeling, along with YAP1 activation in the inflammation‐associated EAP groups.

### 2.3 LPS Enhances the Expression and Nuclear Translocation of YAP1 in BPH‐1 and WPMY‐1 Cells

**Figure 3:**
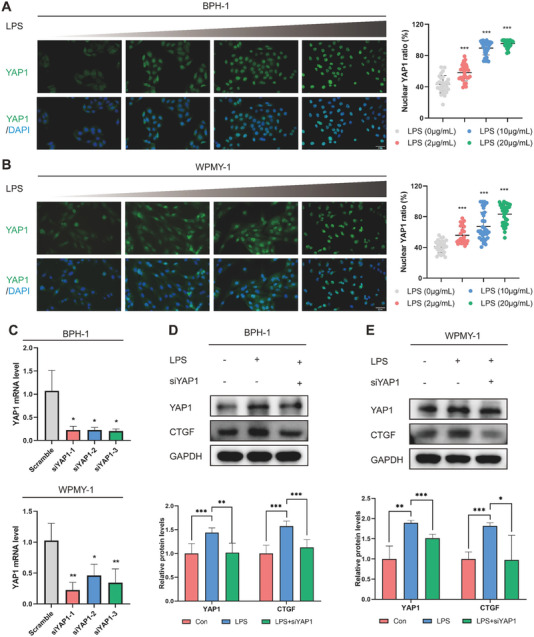
LPS promotes YAP1 nuclear translocation in human prostatic epithelial cell BPH‐1 and stromal cell WPMY‐1. A,B) Representative IF images revealing the effects of lipopolysaccharide (LPS) on the expression and cell localization of YAP1 in the prostatic epithelial cell BPH‐1 and stromal cell WPMY‐1 (*n* ≥ 24 cells were assessed). C) RT‐PCR analysis to determine the inhibitory efficiencies of siRNAs targeting YAP1 (siYAP1). D,E) Immunoblotting analysis to examine the inhibitory effects of siYAP1 on LPS‐induced YAP1/CTGF expression. Data were presented as mean ± SD of 3 to 6 independent experiments. One‐way ANOVA followed by Dunnett's post‐hoc test was used for A,B) to measure statistical significance in comparison to control group. Two‐tailed Student's *t*‐test was used for C–E). *p < 0.05, ** *p* < 0.01, *** *p* < 0.001.

The human prostatic hyperplasia epithelial cell BPH‐1 and stromal cell WPMY‐1 were treated with lipopolysaccharide (LPS) to induce inflammatory responses. As the LPS concentration increased, the expression of YAP1 gradually rose, accompanied by a cytoplasm‐to‐nucleus translocation (**Figure** **3**A,B). LPS induced the upregulation of YAP1/CTGF and reduced the inactivating phosphorylation modification of YAP1 (S127A) in a concentration‐dependent manner in the BPH‐1 and WPMY‐1 cells. Notably, LPS exposure activated YAP1 activity by initiating its nuclear translocation (Figure S1A,B, Supporting Information). Then we designed three pairs of siRNAs (siYAP1) to knockdown the expression of YAP1. The first sequence with the highest knockdown efficiency was selected for subsequent experiments (Figure 3C). The results implied that siYAP1 could significantly abrogate LPS‐induced YAP1/CTGF upregulation in the BPH‐1 and WPMY‐1 cells (Figure 3D,E).

### 2.4 YAP1 Silencing Attenuates Cell Survival, Phenotype Conversion and ECM Production

**Figure 4:**
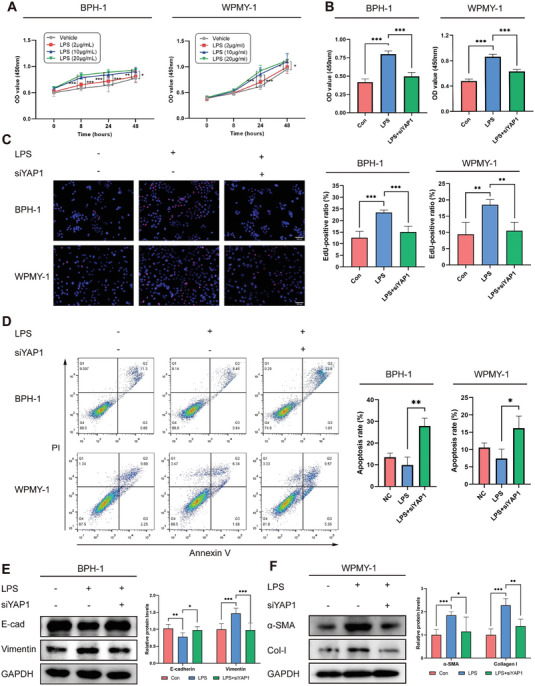
Knockdown of YAP1 influences cell survival, phenotype transition, and ECM generation. A) CCK‐8 assay to determine cell viability of BPH‐1 and WPMY‐1 cells treated with different concentration of LPS during varying incubation periods. B) CCK‐8 assay to determine cell viability of cells transfected with siYAP1. C) Edu assay to discover proliferating cell, and the ratio was calculated through dividing the number of positive cells by the total number of cells. D) Annexin V/PI staining followed by flow cytometry to detect apoptotic cells. E,F) Immunoblotting analysis to evaluate the occurrence of EMT in BPH‐1 cell and ECM generation in WPMY‐1 cell. Data were presented as mean ± SD of 3 to 6 independent experiments. Two‐way ANOVA followed by Dunnett's post‐hoc test was used for A) to measure statistical significance in comparison to control group. Two‐tailed Student's *t*‐test was used for B−F). *p < 0.05, ** *p* < 0.01, *** *p* < 0.001.

As the concentration of LPS increased, the cell viabilities of both BPH‐1 and WPMY‐1 cells significantly increased across various incubation periods (**Figure** **4**A). However, the proliferative effect of LPS was prohibited after siYAP1 treatment (Figure 4B). Upon LPS stimulation, a dose‐dependent increase in the Edu^+^ proliferating cell ratio was observed (Figure S2, Supporting Information), but the ratio was dramatically reduced in the presence of siYAP1 (Figure 4C). In response to LPS stimulation, there was a downregulation of pro‐apoptotic protein Bax, an upregulation of anti‐apoptotic protein Bcl‐2, and a reduction of Bax/Bcl‐2 ratio observed in the BPH‐1 and WPMY‐1 cells (Figure S3, Supporting Information). However, YAP1 silencing accelerated the apoptosis process, characterized by the suppression of Bcl‐2 and the enhancement of Bax (Figure S4A,B, Supporting Information). Similarly, following siYAP1 treatment, the proportion of apoptotic cells was significantly increased in both cells (Figure 4D), confirming that YAP1 suppression exerts a dual influence on cell survival by not only impeding proliferation but also promoting apoptosis.

LPS promoted the process of EMT in BPH‐1 cell, as evidenced by the loss of epithelial marker E‐cadherin and the acquisition of mesenchymal marker Vimentin (Figure S5A, Supporting Information). In addition, it also expedited the generation of ECM proteins in WPMY‐1 cell, characterized by the upregulation of α‐SMA and the production of collagen I (Figure S5B, Supporting Information). Encouragingly, knockdown of YAP1 using siYAP1 significantly reversed the EMT process of BPH‐1 cell and abrogated the ECM generation of WPMY‐1 cell (Figure 4E,F).

### 2.5 LPS Promotes Cytoskeleton Remodeling and YAP1 Upregulation via Activating the RhoA/ROCK1 Pathway

**Figure 5:**
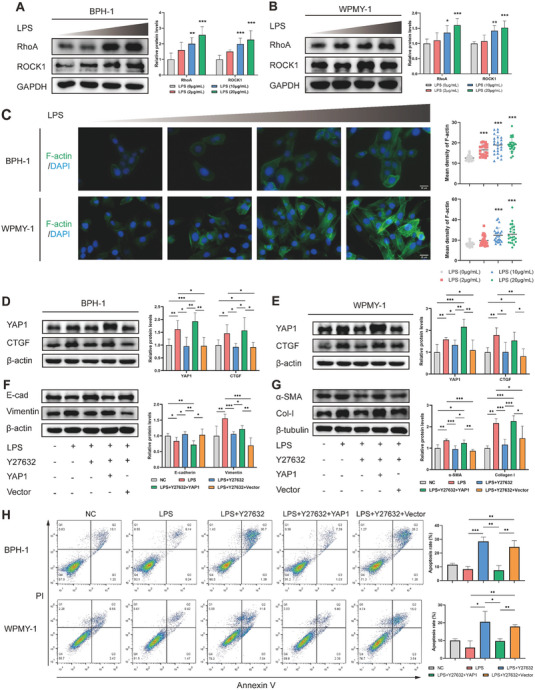
LPS activates RhoA/ROCK1 pathway to induce cytoskeleton remodeling and YAP1 expression. A,B) Immunoblotting analysis showing the expression of RhoA and ROCK1 after LPS stimulation. C) Representative images of phalloidin staining to investigate the cytoskeleton architecture (*n* ≥ 24 cells were assessed). D,E) Immunoblotting analysis showing the expression of YAP1/CTGF in cells treated with a ROCK inhibitor (Y27632) and an ectopic YAP1 expression vector (YAP1). F,G) Immunoblotting analysis to explore the influences of Y27632 and YAP1 vector on EMT process in BPH‐1 cell and ECM generation in WPMY‐1 cell. H) Flow cytometry to evaluate the potential influence of the ROCK1/YAP1 axis on cell apoptosis. Data were presented as mean ± SD of 3 to 6 independent experiments. One‐way ANOVA followed by Dunnett's post‐hoc test was used for A–C) to measure statistical significance in comparison to control group. Two‐tailed Student's *t*‐test was used for D−H). *p < 0.05, ** *p* < 0.01, *** *p* < 0.001.

As mentioned in a previous section, the RhoA/ROCK1 pathway was activated in the prostate tissue of the EAP model compared to that of NC or TI group (Figure 2D). Analysis of the GEPIA2 (http://gepia2.cancer‐pku.cn/) database^[^ ^16^ ^]^ further revealed a strong positive correlation between YAP1 and RhoA or ROCK1 in the normal prostate samples based on the transcriptome data from TCGA and GTEx databases (Figure S6A–D, Supporting Information). Moreover, LPS also facilitated the activation of the RhoA/ROCK1 pathway in a dose dependent manner (**Figure** **5**A,B), and induced F‐actin polymerization, manifested as enhanced stress fiber formation (Figure 5C), in the BPH‐1 and WPMY‐1 cells.

The dynamic reorganization of the actin cytoskeleton is intricately regulated by the RhoA/ROCK1 pathway.^[^ ^17^ ^]^ The aligning of cytoskeletal architecture, notably the formation of stress fibers, influences the nuclear entry of YAP1.^[^ ^18^ ^]^ To elucidate whether the RhoA/ROCK1 pathway regulates YAP1 activity, cells were treated with the ROCK inhibitor Y27632 and transfected with the YAP1 expression plasmid or empty vector. As a result, Y27632 effectively attenuated LPS‐induced YAP1/CTGF upregulation, which was rescued by YAP1 overexpression (Figure 5D,E). Additionally, Y27632 blunted inflammation‐induced EMT switch and ECM generation, and the effects were distinctly reversed after YAP1 overexpression (Figure 5F,G). Pretreatment with Y27632 contributed to an augmentation of the Bax/Bcl‐2 ratio, thus accelerating the cell apoptosis process, whereas YAP1 overexpression yielded converse outcomes (Figure 5H; Figure S7, Supporting Information). These findings suggest that LPS‐activated RhoA/ROCK1 pathway triggers actin polymerization and YAP1 nuclear transduction, thereby promoting cell survival and fibrosis.

### 2.6 YAP1‐TEAD1 Interaction Drives Inflammation‐Associated BPH Progression

**Figure 6:**
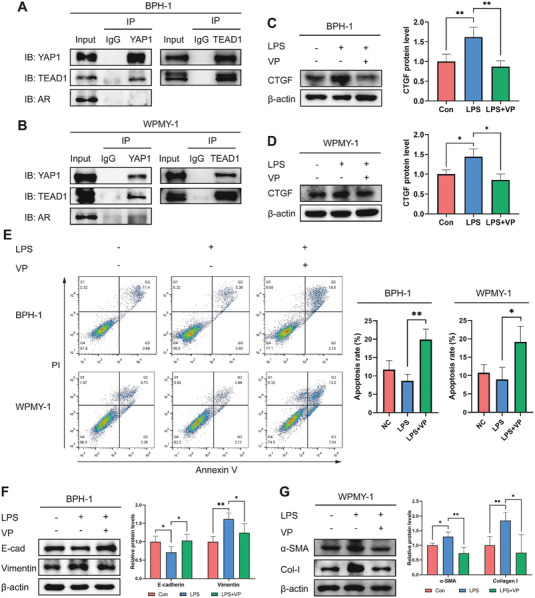
YAP1‐TEAD1 interaction drives the development of inflammation‐associated BPH. A,B) Reciprocal Co‐IP and immunoblotting analysis to explore the protein‐protein interaction between YAP1 and transcriptional factor TEAD1 or AR. C,D) Immunoblotting analysis to examine the inhibitory effect of YAP1‐TEAD inhibitor (VP) on CTGF expression. E) Annexin V/PI staining followed by flow cytometry to recognize apoptotic cells. F,G) Immunoblotting analysis showing expression of EMT and ECM markers in the VP‐treated cells. Data were presented as mean ± SD of 3 to 4 independent experiments. Two‐tailed Student's *t*‐test was used for C–G). *p < 0.05, ** *p* < 0.01.

YAP1 cannot bind to genomic DNA directly, but instead interacts with various transcription factors, and the well‐characterized ones are the TEAD family.^[^ ^12b^ ^]^ Additionally, the interaction between YAP1 and androgen receptor (AR) plays a pivotal role in driving the transition from an androgen‐responsive state to a castration‐resistant state in prostate cancer.^[^ ^19^ ^]^ To further identify the transcriptional regulatory mechanism of YAP1, we conducted reciprocal co‐immunoprecipitation (Co‐IP) experiments and revealed that YAP1 formed a transcriptional complex with TEAD1, but not with AR, in the BPH‐1 and WPMY‐1 cells (**Figure** **6**A,B). The FDA‐approved drug verteporfin (VP), a well‐recognized YAP1‐TEAD binding inhibitor, performed an appreciable inhibitory effect on the expression of their regulatory gene CTGF (Figure 6C,D). The disruption of YAP1‐TEAD1 interaction using VP significantly accelerated cell apoptosis (Figure 6E) through upregulating Bax and downregulating Bcl‐2 in the BPH‐1 and WPMY‐1 cells (Figure S8, Supporting Information). Pretreatment with VP prevented the EMT process of BPH‐1 cells and reduced ECM protein generation of WPMY‐1 cells (Figure 6F,G).

### 2.7 Matrix Stiffness Positively Regulates Actin Polymerization and YAP1 Nuclear Translocation

**Figure 7:**
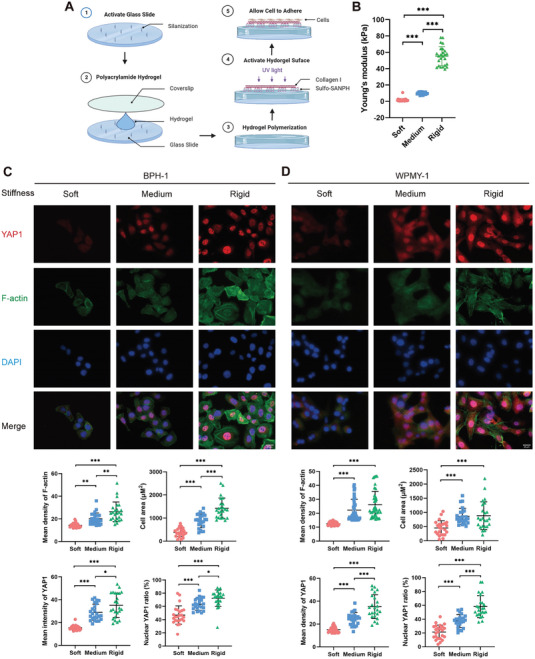
Matrix stiffness positively regulates actin polymerization and YAP1 nuclear translocation. A) Schematic diagram of the synthesis strategies for polyacrylamide hydrogels with varying stiffness. B) Determination of the Young's moduli utilizing atomic force microscope. C,D) Phalloidin staining to investigate the cytoskeleton remodeling and cell morphology by determining average intensity of F‐actin and cell area. IF analysis to unveil the expression and cellular location of YAP1 by determining average intensity and nuclear ratio of YAP1 (*n* ≥ 24 cells were assessed). Data were presented as mean ± SD. One‐way ANOVA followed by Tukey's multiple comparison test was used for C,D). *p < 0.05, ** *p* < 0.01, *** *p* < 0.001.

Extracellular mechanical cue can regulate cytoskeleton and thus influence various cell processes, such as cell growth, migration, and phenotype conversion.^[^ ^12b^ ^]^ Notably, we have just found that inflammation mediated phenotype conversion and secretion of ECM proteins through the activation of YAP1, which led to the enhancement of extracellular mechanical stiffness. Thus, to mimics the transition of the extracellular microenvironment from a normal to a fibrosis state, cells were seeded on polyacrylamide hydrogels with varying degrees of stiffness (**Figure** **7**A,B). The fluorescence intensity of F‐actin and the cell spreading area were affected by the stiffness of the hydrogel. Compared to cells grown on the soft hydrogel, cells grown on the medium or rigid hydrogel had more abundant and denser F‐actin fiber, and the cell morphology appeared more elongated and extended. Meanwhile, increasing matrix stiffness led to a significant upregulation in YAP1 protein level and its translocation into the nucleus (Figure 7C,D). F‐actin intensity was positively associated with YAP1 intensity as well as nuclear YAP1 ratio (Figure S9, Supporting Information), indicating that the mechanical stiffness of the ECM promotes YAP1 nuclear accumulation through the induction of actin polymerization.

### 2.8 Synergistic Promotion of YAP Activity by Inflammatory and Mechanical Cues

**Figure 8:**
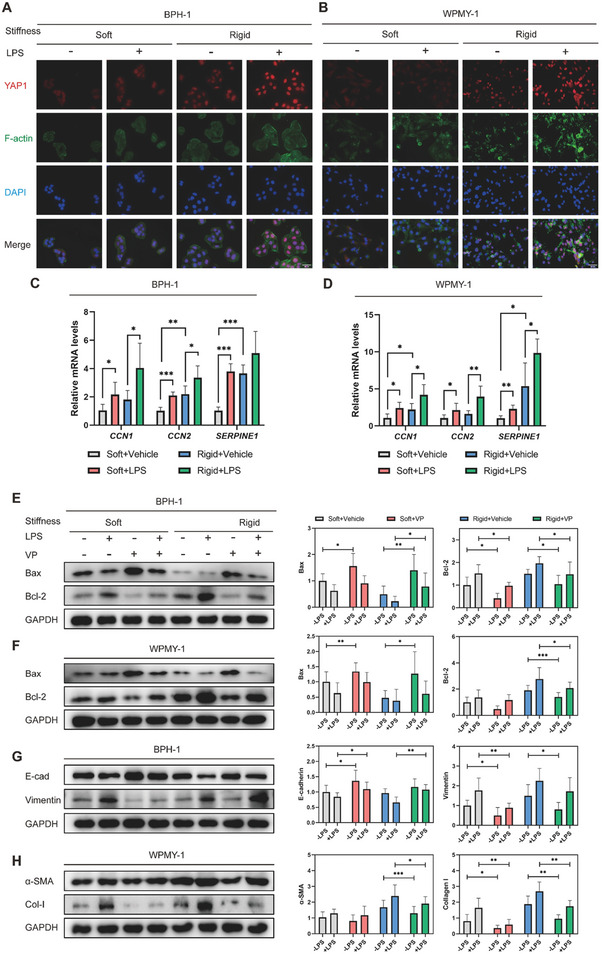
Synergistic enhancement of YAP1 activity by inflammatory and mechanical stimuli. A,B) Representation images of phalloidin and IF staining showing cytoskeleton alignment and YAP1 localization. C,D) RT‐PCR analysis to determine the YAP1 downstream gene expression pattern. E,F). Immunoblotting analysis to evaluate whether YAP1 activity is required for apoptosis resistance, considering the synergetic influences of inflammatory and mechanical cues. G,H) Immunoblotting analysis to evaluate the significance of YAP1 activity in facilizing EMT process in BPH‐1 cell and ECM production in WPMY‐1 cell when the combined influences of inflammatory and mechanical cues was taken in account. Data were presented as mean ± SD of 5 to 6 independent experiments. Two‐tailed Student's *t*‐test was used for C−H). *p < 0.05, ** *p* < 0.01, *** *p* < 0.001.

Given that both physical and biochemical signals enhance YAP1 transcriptional activity, IF analysis was applied to explore whether these cues have synergistic effects on actin alignment and YAP1 localization. The results implied that heightened matrix stiffness induced actin polymerization and YAP1 nuclear translocation, and the addition of LPS further amplified these effects (**Figure** **8**A,B). Moreover, inflammatory and mechanical cues synergistically enhanced YAP1 activity, leading to an increased expression of downstream genes (*CCN1*, *CCN2*, *SERPINE1*) (Figure 8C,D).

Furthermore, the addition of LPS inhibited cell apoptosis in the BPH‐1 and WPMY‐1 cells by upregulating Bax and downregulating Bcl‐2 expression, irrespective of matrix stiffness. However, the cell apoptosis process was reactivated by applying YAP1 inhibitor VP (Figure 8E,F). Likewise, the synergistic induction of EMT conversion in BPH‐1 cells and ECM generation in WPMY‐1 cells, triggered by mechanical and inflammatory cues, were effectively reversed in the presence of VP treatment (Figure 8G,H). Taken together, these findings suggest that YAP1 activation under mechanical and inflammatory stimuli is a determinant of prostatic cell apoptosis and fibrosis.

## 3 Discussion

Accumulating evidence has reported that inflammation‐caused tissue damage and wound healing provoke the native cells or mesenchymal stem cells to secrete a diverse array of pro‐inflammatory mediators and growth factors, allowing epithelial and stromal cells to uncontrolled proliferate.^[^ ^3^, ^20^ ^]^ Tissue fibrosis secondary to inflammation is characterized by active EMT, myofibroblast differentiation, excessive ECM deposition, and reinforced mechanical stiffness.^[^ ^8^, ^21^ ^]^ Prostatic fibrosis contributes to ECM remodeling, impairing tissue elasticity and compliance, thereby aggravating LUTS performance of BPH patients.^[^ ^8^, ^22^ ^]^ In a previous study, we showed that EAP rat model exhibited obvious BPH pathological features, such as tissue hyperplasia, reactive stroma accumulation and ECM deposition. However, the mechanism underlying inflammation‐induced BPH progression is not completely understood.

Despite the role of YAP1 in prostate cancer is well‐established, it is unclear whether it is also involved in BPH progression. Both physical and biochemical signals can motivate YAP1 to translocate into the nucleus, where YAP1 interacts with various transcriptional factors to initiate the expression of genes involved in cell survival, tumorigenesis, and organ fibrosis.^[^ ^12^ ^]^ Notably, pathological fibrotic conditions trigger ECM remodeling and alter the mechanical properties of the tissue matrix. YAP1 can act as a mechanical transducer and sense alterations in ECM stiffness, eventually exacerbating the fibrotic process.^[^ ^23^ ^]^ For instance, ECM stiffening facilitated the formation of YAP1‐Smad3 complex, which hastened the proliferation of bladder smooth muscle cells (SMC) by activating the MAPK/ERK signaling, thereby worsening bladder fibrosis.^[^ ^24^ ^]^ Moreover, the N‐cadherin/YAP1 complex was implicated in nucleus pulposus cell ferroptosis in the context of ECM‐mediated mechanotransduction.^[^ ^25^ ^]^

The Hippo/YAP1 pathway is also a prominent regulator of the immune responses. LPS was responsible for the upregulation of YAP1 in Kupffer cells, and the YAP1‐TEAD complex promoted the production of pro‐inflammatory mediators (MCP‐1, TNF‐α, and IL‐6) and pro‐fibrotic molecules (α‐SMA, collagen I).^[^ ^26^ ^]^ The present study showed that YAP1 expression was significantly increased in the prostate of the EAP model rats and the inflamed‐BPH patients. YAP1 accumulated in the nucleus and led to the expression of downstream ECM remodeling‐associated genes (*CCN1*, *CCN2*, *SERPINE1*). In addition, YAP1 expression was positively associated with the collagen fiber deposition and IPSS score, but negatively correlated with TPV. This is not surprising given that all tissue specimens were obtained from patients who underwent transurethral resection of the prostate (TURP) surgery. Such patients with relatively low prostate volume may desire for surgical intervention due to the YAP1‐associated prostate fibrosis and aggravated LUTS performance. Meanwhile, in vitro experiments also verified that LPS exposure promoted YAP1 translocation into the nucleus, where it bound to TEAD1 and initiated gene transcription of CTGF.

The balance of proliferation and apoptosis in cells is the basis of growth homeostasis, while any disruptions in this balance may contribute to the progression of BPH.^[^ ^27^ ^]^ We found that genetic silencing of YAP1, or pharmacological inhibition of YAP1‐TEAD interaction, significantly reduced cell proliferation and induced apoptosis of the BPH‐1 and WPMY‐1 cells, as evidenced by decreased cell viability and Edu^+^ proliferating cell numbers, and increased Bax/Bcl‐2 ratio and apoptotic cell numbers. The occurrence of EMT can cause the polarized epithelial cells to gain a mesenchymal phenotype, characterized by apoptosis resistance and active ECM synthesis.^[^ ^28^ ^]^ What else, stromal cells have the potential to differentiate into easily‐contractile α‐SMA‐positive myofibroblasts, which are prone to secrete high amounts of ECM proteins such as collagen I/III, ultimately resulting in the replacement of normal tissue by stiff and low‐compliant fibrotic tissue.^[^ ^22^, ^29^ ^]^ We found that the inhibition of YAP1 reversed LPS‐induced EMT switch of BPH‐1 cells and ECM proteins synthesis of WPMY‐1 cells. These findings indicated that targeting YAP1 could suppress cell growth and fibrosis, achieved by inhibiting proliferation, accelerating apoptosis, reversing EMT and decreasing ECM production, thereby preventing BPH progression.

**Figure 9:**
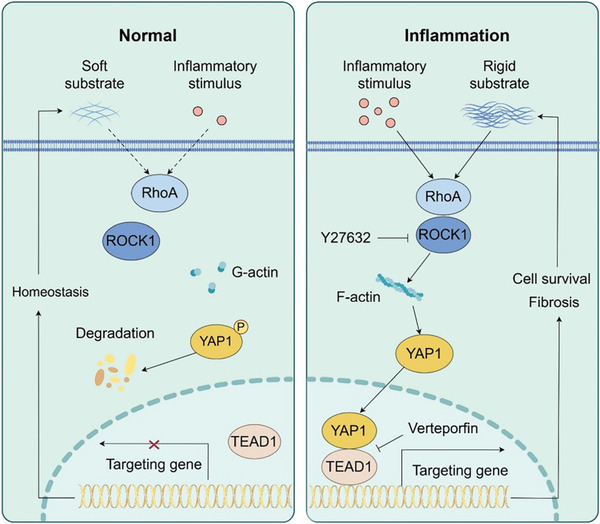
Schematic diagram depicting that inflammatory and mechanical cues synergistically promote prostatic cell survival and fibrosis through activating the RhoA/ROCK1/F‐actin/YAP1 axis.

The aforementioned findings suggested that chronic inflammation triggered the activation of YAP1, leading to prostatic hyperplasia, epithelial cell EMT progress, and stromal cell ECM protein synthesis, all of which tend to reinforce tissue stiffness. Emerging evidence implies that YAP1 functions as a sensor and responder to mechanical signals. Mechanical signals can activate small molecules Rho GTPase, such as RhoA/ROCK1, to modify the cytoskeleton and thus motivate YAP1 nuclear entry to participate in various biological processes including cell proliferation, EMT, and tissue remodeling.^[^ ^11^, ^15^, ^18^ ^]^ The present study revealed that inflammation activated RhoA/ROCK1 signaling and induced F‐actin assembly. The application of ROCK inhibitor Y27632 reduced the expression of YAP1/CTGF, and thus prevented cell survival, EMT progression, and ECM synthesis. However, the inhibitory effects of Y27632 were significantly reversed by YAP1 overexpression. These findings suggested that inflammation‐activated RhoA/ROCK1/F‐actin pathway is required for YAP1 activation. To further evaluate the direct impact of mechanical cue on YAP1 activation, the prostatic cells were grown on hydrogel matrices of varying stiffnesses. The results showed that a rigid matrix stiffness could induce F‐actin polymerization and YAP1 nuclear accumulation. Noteworthily, the combined physical and biochemical cues can exert a substantial influence on cell behavior and phenotype switching.^[^ ^30^ ^]^ For example, a stiff substrate triggered the differentiation of neural crest stem cells (NCSCs) into vascular smooth muscle cells (SMCs) in the presence of TGF‐β1.^[^ ^30b^ ^]^ In the present study, inflammatory and mechanical cues synergistically activated YAP1, while the suppression of YAP1 activity impaired the synergistic effects of these cues on cell survival and fibrogenesis. Collectively, these results suggest that inflammation promotes YAP1 expression and mediates prostatic cell proliferation, apoptosis resistance, EMT transformation, and ECM protein synthesis, ultimately leading to mechanical stiffness reinforcement. The reinforced mechanical stiffness, working in synergy with inflammatory cues, reactivates YAP1 via triggering cytoskeleton remodeling. This forms a positive feedback loop that continuously propels the progression of prostatic hyperplasia and fibrosis (**Figure** **9**).

However, some limitations still existed in this study. First, given the multitude of pathogenic factors contributing to prostatic inflammation, LPS alone may not entirely simulate the complex in vivo environment. Therefore, it is essential to conduct animal experiments to verify whether targeting YAP1 is sufficient to treat inflammation‐associated BPH model. Second, this study found that inflammation, as an initiating factor, promotes fibrosis progression through the establishment of a positive feedback loop involving RhoA/ROCK1/F‐actin/YAP1. Further experiments are required to determine whether YAP1‐TEAD1 transcriptionally activates the pro‐inflammatory factors, thereby maintaining and amplifying the local inflammatory and mechanical signals.

## 4 Conclusion

This study first highlights the prominent role of YAP1 in orchestrating cell survival, EMT, and ECM protein synthesis during the progression of inflammation‐associated BPH. The fibrotic microenvironment strengthens mechanical cue, which, in turn, induced cytoskeleton remodeling and YAP1 reactivation, thereby forming a positive feedback loop to promote prostatic hyperplasia and fibrosis. Inhibition of YAP1 may represent a promising strategy to terminate the vicious cycle between prostatic inflammation and fibrosis.

## 5 Experimental Section

### Human Prostatic Specimen

Human prostate specimens were acquired from BPH patients who were undergoing TURP surgery. Based on histological performance of hematoxylin & eosin (HE) staining, the specimens without any signs of inflammation lesion in all fields were categorized as BPH group (*n* = 15), and those with obvious inflammatory cell infiltration in at least three randomly selected fields (100×) were categorized as inflamed‐BPH group (*n* = 15). The specimens with scattered inflammatory cells or inflammation foci observed in only a few fields were excluded. One part of each tissue was embedded in paraffin and used for subsequent histopathological detection and immunostaining, while the remaining part was preserved in liquid nitrogen for molecular biology analysis. The clinical information, including age, PSA level, IPSS was recorded. The age distribution was similar between the BPH and inflamed‐BPH groups. The TPV was calculated using the prolate ellipsoid formula (TPV = 0.5233 × transverse length × cranial caudal length × anteroposterior length).^[^ ^31^ ^]^ All experiments involving human specimens were approved by the Ethics Committee of Tongji Hospital, and informed consent was obtained from all patients (approval no. TJ‐IRB20211113).

### Experimental Animals

Twenty‐four male Wistar rats (8 week old, 250–300 g) were provided by Vital River company and housed in a quiet environment with a regular 12 h light‐dark cycle. After one week of acclimatization, the animals were randomly categorized into one of three groups: 1) NC; 2) TI; 3) EAP. The rats in the NC group underwent sham operation and were injected with an equal volume of PBS buffer. For the TI group, the rats were castrated to eliminate the influence of endogenous testosterone, and then received daily subcutaneous administration of 5 mg kg^−1^ testosterone propionate (Sigma–Aldrich, MO, USA).^[^ ^32^ ^]^ The rats in inflammation‐associated EAP model was established by intradermally injections of a mixture of prostatic homogenate and complete Freund's adjuvant on day 0 and day 30.^[^ ^5a^ ^]^ The prostates from all groups were dissected and embedded in paraffin or preserved in liquid nitrogen on day 45. All animal operations were approved and supervised by the Ethics Committee of the Laboratory Animal Center of Tongji Hospital (approval no. TJH‐202101005).

### Histological Examination and Scoring System

The tissue specimens were fixed in 4% paraformaldehyde, dehydrated in a series of graded ethanol, embedded in paraffin, and then sectioned into 5 µm‐thick slices. The sections were stained with HE and Masson dyes to observe the histopathological features and collagen fiber deposition, respectively. All images were captured under a light microscope (SDPTOP, Shanghai, China). The Histoscore protocol, which considers epithelial morphology and layers, stroma abundant, cell polarity, and other factors, was applied to compare the histopathological features of the three groups.^[^ ^33^ ^]^ The histopathological findings were scored in a blinded manner.

### Immunohistochemistry and Immunofluorescence Observation

For Immunohistochemistry (IHC) analysis, the sections were heated in citrate buffer for antigen retrieval, treated with H_2_O_2_ to quench the endogenous peroxidases, and then incubated with primary antibodies against YAP1 or PCNA overnight. The sections were then probed with HRP‐conjugated secondary antibody, developed using DAB, and counterstained with hematoxylin. The cells with positive YAP1 or PCNA reactions were analyzed by ImageJ software. For IF analysis, the sections were sequentially incubated with anti‐YAP1 primary antibody and FITC‐labelled secondary antibody. To determine YAP1 localization, DAPI staining was applied to outline the nucleus border and distinguish the nucleus region from the cytoplasm region. The average fluorescence intensities and nucleus/cytoplasm ratios were measured using ImageJ software.

### Hydroxyproline Concentration Assessment

An aliquot (50 mg) of prostatic specimen was weighed and homogenized in 500 µL 0.9% saline solutions. Hyp, a preferable collagen content marker, was quantified via measuring the conversion of dimethyl‐aminobenzaldehyde to a colorimetric product, following the standard manufacturing procedure (Nanjing Jiancheng Bioengineering Institute, Nanjing, China).

### Cell Lines and Treatments

The human prostatic hyperplasia epithelial cell line BPH‐1 was acquired from Leibniz Institute DSMZ, while the human normal prostatic stromal cell line WPMY‐1 was acquired from American Type Culture Collection (ATCC). Depending on the experimental requirements, the cells were treated with LPS (Sigma–Aldrich, MO, USA), the specific YAP1‐TEAD interaction inhibitor VP (2 µm), or the ROCK kinase activity inhibitor (Y27632, 5 µm).

### Cell Transfection

The cells were transfected with siRNA targeting YAP1 (siYAP1) using RNAiMAX reagent (Invitrogen, CA, USA) following the manufacturer's instructions. The sequence of siRNA (siYAP1) with the best inhibitory efficiency was selected for further study. Non‐targeting siRNA (Scramble) was applied as the negative control. In another experiment, the cells were transfected with an ectopic overexpression plasmid (YAP1, purchased from MiaoLing Plasmid Platform). An empty vector (Vector) was applied as the negative control.

### Immunoblotting Analysis

Total protein was isolated using RIPA lysis buffer. Equal amounts of total protein from each sample were separated by SDS‐polyacrylamide gel electrophoresis (SDS‐PAGE) and then transferred onto a PVDF membrane (Millipore, MA, USA). The membrane was incubated with 3% bovine serum albumin (BSA) solution, followed by incubation with primary antibody at 4 °C overnight. The membrane was immunoreacted with HRP‐conjugated secondary antibody for 1 h the following day. After treating with ECL chemiluminescence substrate, the band of targeted protein was exposed via a chemiluminescence imager (Bio‐Rad, CA, USA). The results were normalized and then quantified via ImageJ software. The antibodies’ information was listed in Table S2 (Supporting Information).

### Real‐Time Quantitative PCR

TRIzol Reagent (Takara, Dalian, China) was applied to isolate total RNA following the operation protocol. The total RNA was reversely transcribed into cDNA by PrimeScript RT Master Mix (Takara, Dalian, China), and then mRNA level was quantified by TB Green Premix Ex Taq II Kit (Takara, Dalian, China). The 2^−△△Ct^ method was utilized to determine the relative gene expression, with the housekeeping gene GAPDH regarded as the normalized reference. The primers’ sequences were listed in Table S3 (Supporting Information).

### Cell Viability Assessment

5 × 10^3^ cells were placed into 96‐well plates with complete culturing medium containing different concentrations of LPS, and cultured for 8, 24, and 48 h. To further assess the influence of YAP1 knockdown on LPS‐induced cell proliferation, cells were pre‐incubated with 50 nM siYAP1 and then stimulated with 20 µg mL^−1^ LPS for 48 h. Finally, 10 µL CCK‐8 solution (Vazyme Biotech, Nanjing, China) was added to each well, and the cells were incubated for 1 h. The absorbance was detected at 450 nm.

### Cell Proliferation and Apoptosis Evaluation

Cells were seeded into culture plates and cultured for 24 h to allow cell attachment. Then proliferating cells were determined using the Edu Cell Proliferation Kit (Beyotime Biotechnology, Shanghai, China), and the positive signals were captured under a fluorescence microscopy (Bio‐Rad, CA, USA). The percentage of proliferating cells was calculated through dividing the number of Edu^+^ cells by the number of Hoechst^+^ cells. Apoptotic cells were directly determined using the Annexin V/PI kit (Vazyme Biotech, Nanjing, China) according to the manufacturer's instructions, and the cells were analyzed by flow cytometry.

### Co‐immunoprecipitation Assay

Whole cell lysates were incubated with either YAP1, TEAD1 or control IgG antibody after setting aside a portion as input. Subsequently, protein A/G magnetic beads (Thermo Fisher Scientific, MA, USA) were used to capture protein‐antibody immune complexes. The immune complexes were denatured through boiling in 1× loading buffer for 10 min. Finally, the supernatants were processed with immunoblotting analysis to validate the interaction between YAP1 and TEAD1 or AR.

### Polyacrylamide Hydrogel Preparation

The polyacrylamide hydrogels with varying stiffness were prepared by adjusting the relative ratio of monomer acrylamide (Acr) and cross‐linker bis‐acrylamide (Bis) as reported previously.^[^ ^34^ ^]^ Briefly, prepolymer solutions were prepared by mixing 40% Acr and 2% Bis in three different ratios, and 10 µL TEMED and 100 µL 10% APS were added to expedite the polymerization process. The mixed solutions were quickly transferred onto silanized glass slides and covered with pre‐activated coverslips. After 15 min of polymerization, the hydrogels were gently detached from the glass slides and placed on a 6‐well plate. The hydrogel surface was treated with crosslinker Sulfo‐SANPAH (Thermo Fisher Scientific, MA, USA) and exposed to ultraviolet light (365 nm), followed by coating with 0.2 mg mL^−1^ collagen I (Yeason, Shanghai, China). The stiffness of the hydrogels was evaluated by calculating the Young's moduli via atomic force microscopy (Bruker, Germany). Force curves were recorded from different indentation tests and Young's moduli were computed by NanoScope software (version 1.9).

### Cell Immunofluorescence and Phalloidin Staining

Cells were fixed with 4% paraformaldehyde for 15 min, permeabilized with 0.1% Triton X‐100 for 20 min, and blocked using 3% BSA for 15 min. Actin cytoskeleton (F‐actin) was visualized by FITC‐labeled phalloidin (Yeason, Shanghai, China) staining, while YAP1 localization was evaluated by IF analysis. Cell nucleus was recognized by DAPI dye. The stress fiber intensity and spread area of F‐actin, as well as the fluorescence intensity and nuclear ratio of YAP1 were quantified via ImageJ software.

### Statistical Analysis

Statistical analysis and graphical visualization were realized via GraphPad Prism 8 software. The data were presented as mean ± SD of at least three individual experiments. Statistical significance was evaluated through either two‐sided unpaired Student's *t*‐test or one‐way ANOVA followed by Dunnett's post‐hoc test. Correlation between two variables was analyzed by either Pearson or Spearman test as appropriate. *p* < 0.05 was considered statistically significant.

## Conflict of Interest

The authors declare no conflict of interest.

## Author Contributions

Z.C. conceived and designed the present study. D.X.L. contributed to formal analysis, data curation, investigation, methodology and manuscript writing. C.C.L., P.Y,W., A.Z., and M.Y.Z. performed the investigation, formal analysis, and result visualization. X.L.W., B.L.D., Z.P.L., and K.C. carried out project administration and manuscript revising. All authors have read and approved the final manuscript.

## Supporting information

Supporting Information

## Data Availability

The data that support the findings of this study are available from the corresponding author upon reasonable request.
