# Supplementary material for: YAP1 Recognizes Inflammatory and Mechanical Cues to Exacerbate Benign Prostatic Hyperplasia via Promoting Cell Survival and Fibrosis
Source: Adv Sci (Weinh). 2023 Dec 4;11(5):2304274. doi: 10.1002/advs.202304274 (PMC10837380; doi:10.1002/advs.202304274)
Supplement: Supplementary file 1 — Supporting Information [file ADVS-11-2304274-s001.pdf]

ADVANCED SCIENCE
Open Access

Supporting Information

for *Adv. Sci.*, DOI 10.1002/advs.202304274

YAP1 Recognizes Inflammatory and Mechanical Cues to Exacerbate Benign Prostatic Hyperplasia via Promoting Cell Survival and Fibrosis

*Dongxu Lin, Changcheng Luo, Pengyu Wei, An Zhang, Mengyang Zhang, Xiaoliang Wu, Bolang Deng, Zhipeng Li, Kai Cui and Zhong Chen**

Supporting Information

YAP1 Recognizes Inflammatory and Mechanical Cues to Exacerbate Benign Prostatic Hyperplasia via Promoting Cell Survival and Fibrosis

*Dongxu Lin, Changcheng Luo, Pengyu Wei, An Zhang, Mengyang Zhang, Xiaoliang Wu, Bolang Deng, Zhipeng Li, Kai Cui, Zhong Chen**

Table S1. Evaluating histopathological feature of rat prostate using histoscore protocol.

Histoscore	NC	TI	EAP
Low-power magnification (100×)			
Luminal shape: regular (1); villous (3); papillary (4);	2.08±0.72	3.46±0.72*	2.46±0.72#
Acinar shape: tubular (1); branched (3); irregular (5)	2.50±0.89	3.88±0.99*	3.33±0.76*,#
Interacinar space: large or moderate (1); back-to-back glands (5)	1.67±1.52	1.83±1.66	2.17±1.86
Stroma: fine (1); abundant (3); fibrosis/severe smooth muscle hyperplasia (5)	1.25±0.68	1.67±0.96	3.83±1.31*,#
High-power magnification (400×)			
Epithelial shape: flattened or cuboidal (1); cylindrical (3); hexagonal (5)	1.75±0.99	2.92±1.10*	2.67±1.27*
Number of layers: mono-, 1 (1); oligo, 2-4 (3); pluri, ＞5 (5)	1.17±0.57	2.92±1.25*	2.96±1.33*
If layer ＞1, then add: focal (3); diffuse (5)	1.04±1.43	3.75±0.99*	3.50±1.44*
Alignment:			
Polar (1); apolar (3)	1.08±0.41	1.83±1.01*	1.25±0.68#
If there is piling up of epithelial cells, then add (3)	0.88±1.39	2.63±1.01*	2.25±1.33*
If there is budding out of epithelial cells into stroma, then add (5)	0.63±1.69	2.63±2.50*	2.50±2.55*
If periacinar clusters of epithelial cells are found, then add (3)	0.25±0.85	1.38±1.53*	2.00±1.45*
If isolated clusters of epithelial cells are found outside acini, then add (5)	0.21±1.02	1.04±2.07	0.83±1.90
Lesion distribution			
Unilobar: isolated (2); multiple (6)	1.08±1.44	5.33±1.52*	4.67±1.93*
Bilobar: isolated (4); multiple (8)	1.83±2.04	4.50±1.35*	4.83±1.66*
Nuclear shape			

Round, regular (1); irregular (5)	1.17±0.82	1.67±1.52	1.67±1.52
Small or large (2); small and large in the same acinus (4)	2.17±0.57	2.42±0.83	2.25±0.68
Mitoses per field: absent, 0 (0); isolated, 1-2 (2); abundant, 3-5 (5); excessive, ＞5 (10)	0.33±0.76	2.79±1.50*	2.58±1.91*
Basement membrane:			
Intact (1); interrupted (5)	1.17±0.82	2.33±1.93*	2.67±2.01*
Thin (1); thick (5)	1.33±1.13	3.33±2.01*	3.50±1.98*
Total score (Histoscore)	23.58±9.15	52.29±6.80*	40.13±8.14*,#

Data were expressed as mean ± SD. *Significant difference from NC group, $P < 0.05$. #Significant difference from TI group, $P < 0.05$.

Table S2. The information of primary antibodies used in this study.

Antibody	Application	Concentration	Company	Catalogue
YAP1	WB, IF, IHC	1:1000 (WB); 1:100 (IF); 1:400 (IHC)	Proteintech Group	13584-1-AP
YAP1	IP	1:20	Santa Cruz Biotechnology	sc-101199
p-YAP1 (S127A)	WB	1:1000	Boster	BM4580
TEAD1	WB, IP	1:1000 (WB); 1:50 (IP)	Abmart	T59103
CTGF	WB	1:1000	Abclonal	A11067
AR	WB	1:1000	Servicebio	GB115311
PCNA	IHC	1:400	Boster	BM0104
E-cadherin	WB	1:1000	Proteintech Group	20874-1-AP
Vimentin	WB	1:1000	Servicebio	GB11192
Collagen I	WB	1:1000	Proteintech Group	14695-1-AP
α-SMA	WB	1:1000	Boster	BM0002
TGF-β	WB	1:1000	Proteintech Group	21898-1-AP
Bax	WB	1:1000	Abmart	T40051
Bcl-2	WB	1:1000	Abmart	T40056
RhoA	WB	1:1000	Proteintech Group	10749-1-AP
ROCK1	WB	1:1000	Proteintech Group	21850-1-AP
GAPDH	WB	1:2000	Proteintech Group	60004-1-Ig
β-actin	WB	1:2000	Abmart	T40104
β-tubulin	WB	1:2000	Abmart	M30109

Table S3. The sequences of RT-PCR primers used in this study.

Gene	Species	Direction	Sequence (5'-3')
CCN1	Human	Forward	ACCGCTCTGAAGGGGATCT
CCN1	Human	Reverse	ACTGATGTTTACAGTTGGGCTG
CCN2	Human	Forward	ACCGACTGGAAGACACGTTTG
CCN2	Human	Reverse	CCAGGTCAGCTTCGCAAGG
SERPINE1	Human	Forward	AGTGGACTTTTCAGAGGTGGA
SERPINE1	Human	Reverse	GCCGTTGAAGTAGAGGGCATT
GAPDH	Human	Forward	TCCCATCACCATCTTCCA
GAPDH	Human	Reverse	CATCACGCCACAGTTTCC
CCN1	Rat	Forward	AGAGGCTTCCTGTCTTTGGC
CCN1	Rat	Reverse	CTCGTGTGGAGATGCCAGTT
CCN2	Rat	Forward	CAGTGTGAAGACCTACCGGG
CCN2	Rat	Reverse	GTAATGGCAGGCACAGGTCT
SERPINE1	Rat	Forward	TTCAAGCTCTTCCGGACCAC
SERPINE1	Rat	Reverse	AATAGAGGGCGTTCACCAGC
GAPDH	Rat	Forward	AGTGCCAGCCTCGTCTCATA
GAPDH	Rat	Reverse	GACTGTGCCGTTGAACTTGC

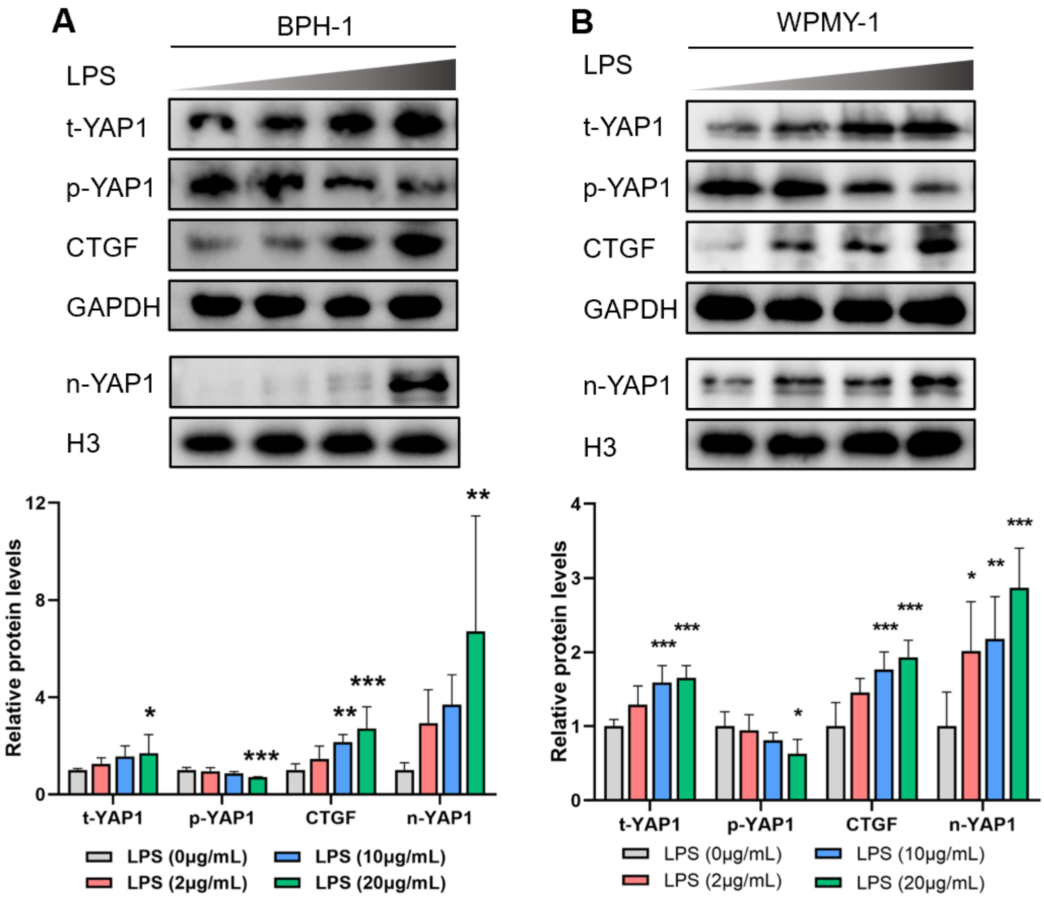

Figure S1. LPS reduces the inactivating phosphorylation of YAP1 at Ser-127 and promoted YAP1 nuclear localization. A, B) Immunoblotting analysis to investigate the impact of LPS on the expression of total-YAP1 (t-YAP1), phospho-YAP1-Ser127 (p-YAP1), CTGF in the whole cell lysate, as well as the expression of nuclear-YAP1 (n-YAP1) in the nuclear lysate. Data were presented as mean ± SD of at least 5 independent experiments. One-way ANOVA followed by Dunnett's post-hoc test was used for A, B) to measure statistical significance in comparison to control group. *$p < 0.05$, ** $p < 0.01$, *** $p < 0.001$.

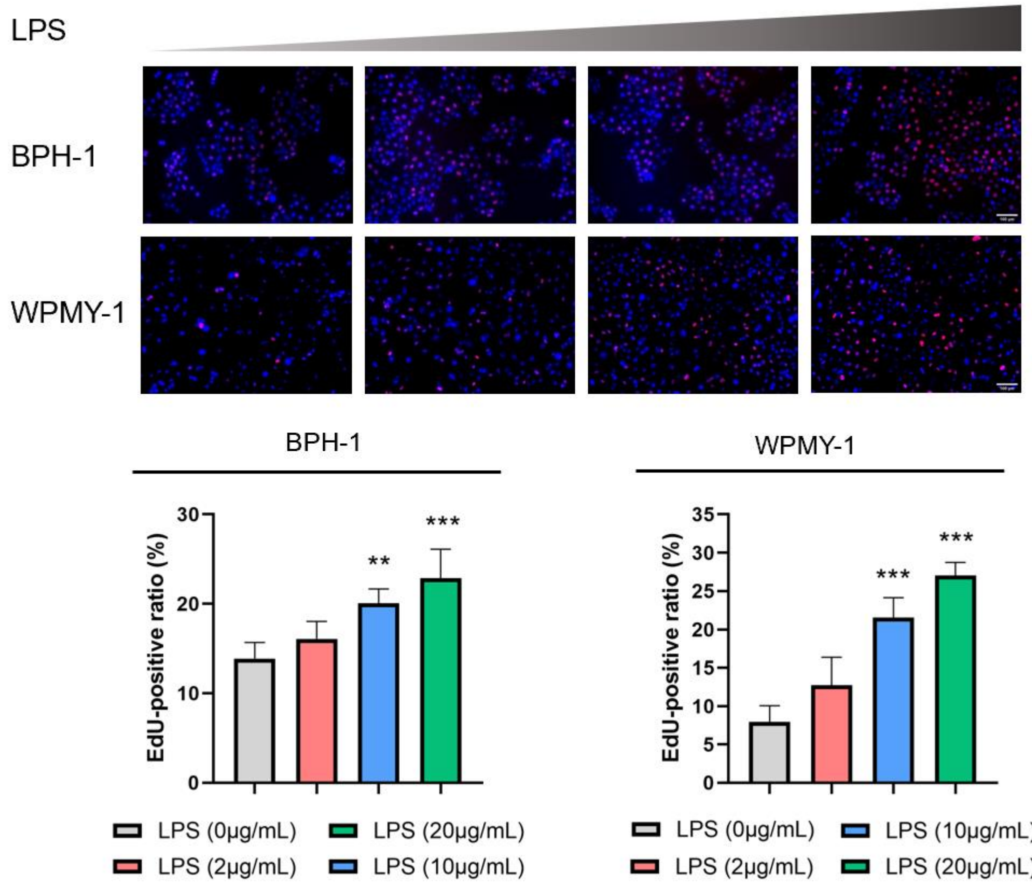

Figure S2. LPS promotes prostatic cell proliferation in a concentration-dependent manner. EdU assay was applied to identify EdU-positive cells. The proliferating cell ratio was calculated through dividing the number of EdU-positive cells by the number of Hoechst-positive total cells. Data were presented as mean ± SD of at least 3 independent experiments. One-way ANOVA followed by Dunnett's post-hoc test was used to measure statistical significance in comparison to control group. ** $p < 0.01$, *** $p < 0.001$.

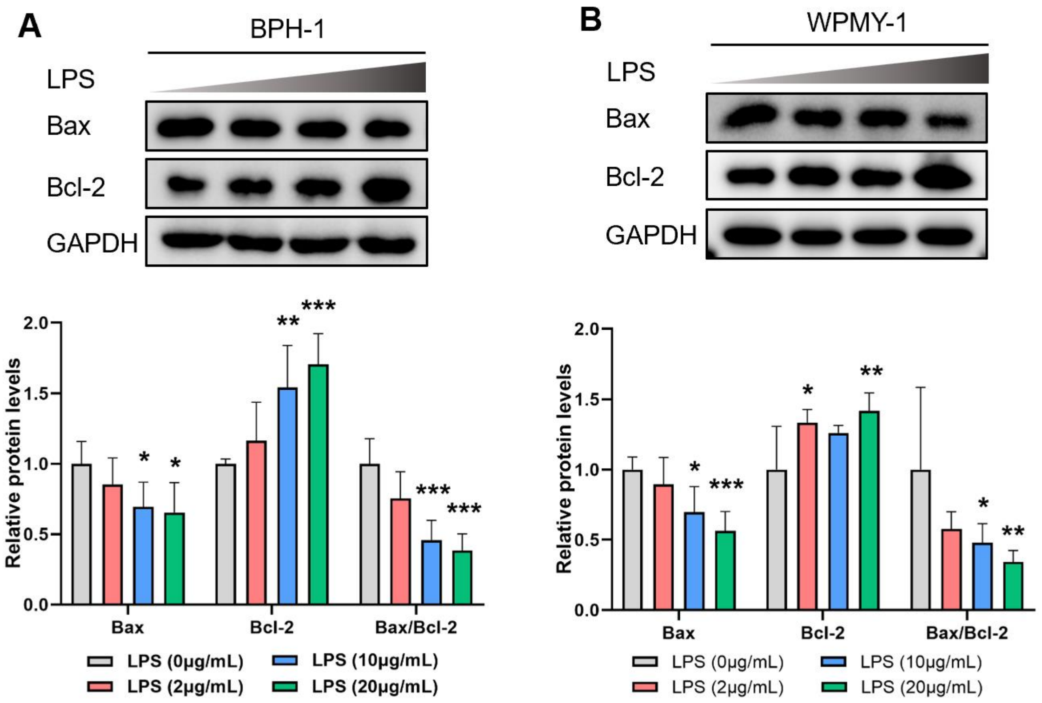

Figure S3. LPS dose-dependently promotes apoptosis resistance in prostatic cells. A, B) Immunoblotting analysis revealed that LPS decreased the expression of the pro-apoptotic protein Bax but increased the expression of the anti-apoptotic protein Bcl-2, leading to a reduced Bax/Bcl-2 ratio in both BPH-1 and WPMY-1 cells. Data were presented as mean ± SD of at least 5 independent experiments. One-way ANOVA followed by Dunnett's post-hoc test was used to measure statistical significance in comparison to control group. ** $p < 0.01$, *** $p < 0.001$.

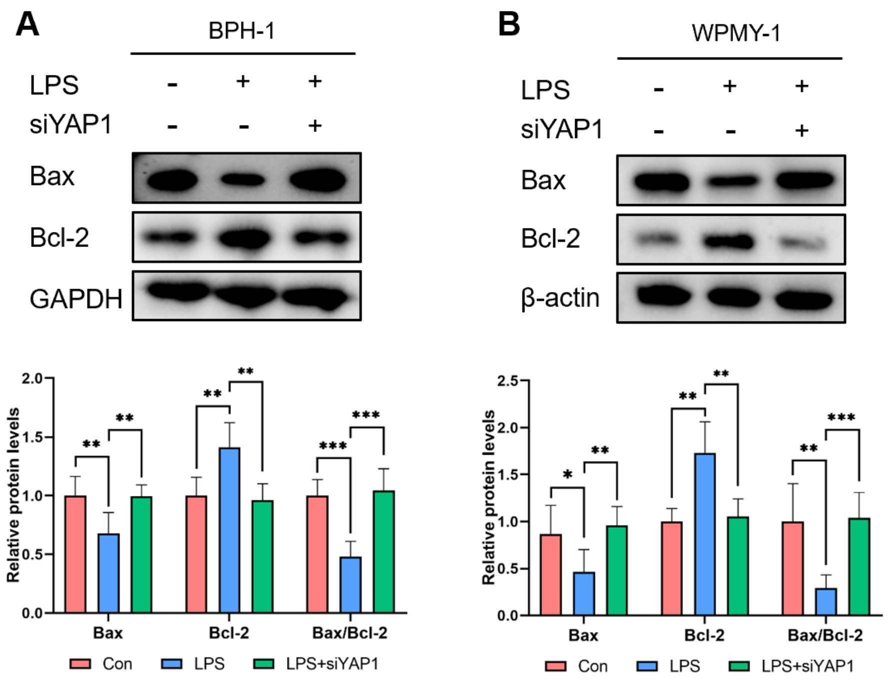

Figure S4. YAP1 silencing participates in the occurrence of apoptosis in prostatic cells. A, B) Immunoblotting analysis to explore the influence of YAP1 knockdown on the expression of apoptosis-regulated proteins Bax and Bcl-2 in the presence of LPS stimulation. Data were presented as mean ± SD of at least 5 independent experiments. Two-tailed Student's *t*-test was used for A, B). $*p < 0.05$, $** p < 0.01$, $*** p < 0.001$.

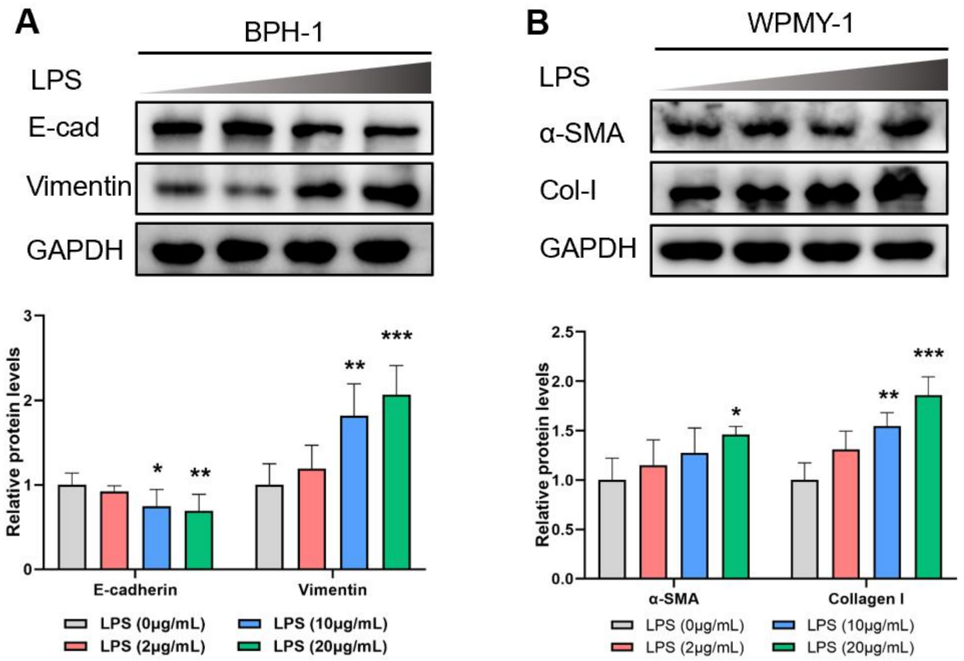

Figure S5. LPS dose-dependently promotes EMT switch of BPH-1 cell and ECM production of WPMY-1 cell. A) Immunoblotting analysis revealed that application of LPS triggered EMT process manifested as decreased E-cadherin expression but increased Vimentin expression. B) Immunoblotting analysis revealed that application of LPS accelerated ECM production characterized by enhanced α-SMA and Collagen I expression. Data were presented as mean ± SD of at least 5 independent experiments. One-way ANOVA followed by Dunnett's post-hoc test was used to measure statistical significance in comparison to control group. * $p < 0.05$, ** $p < 0.01$, *** $p < 0.001$.

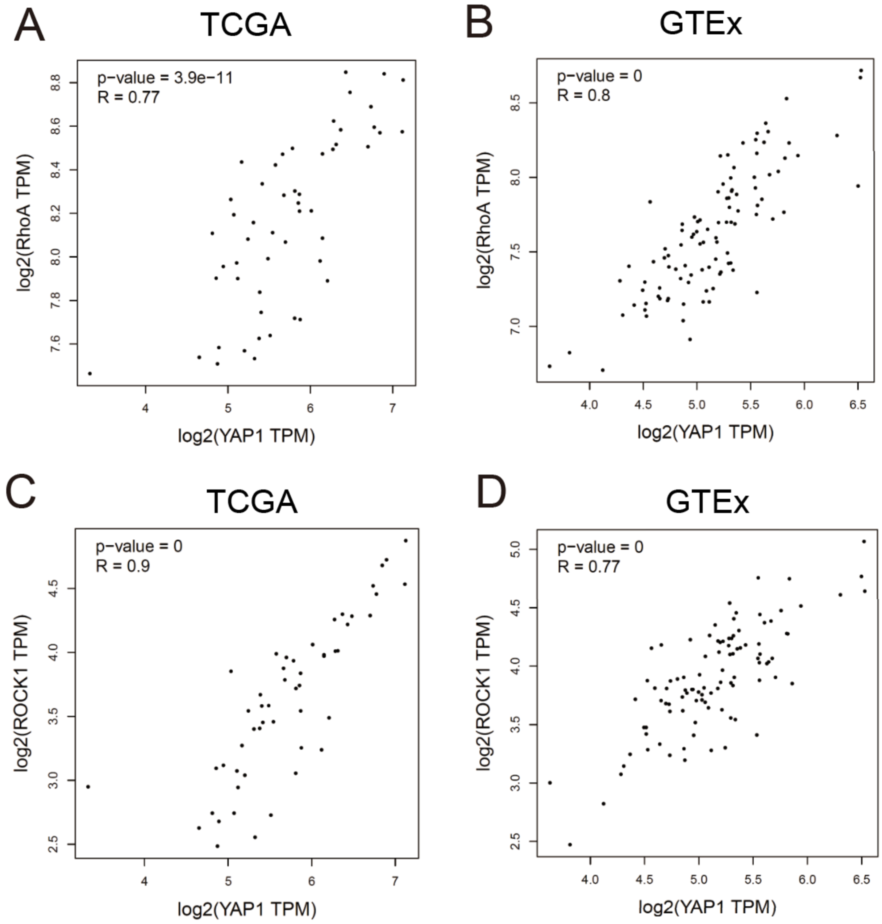

Figure S6. Positive correlation between YAP1 and RhoA or ROCK1 expression in the normal prostate samples. A, B) Correlation analysis of YAP1 and RhoA in the normal prostate samples from TCGA database and GTEx database. C, D) Correlation analysis of YAP1 and ROCK1 in the normal prostate samples from TCGA database and GTEx database. Spearmen correlation analysis was applied to A-D).

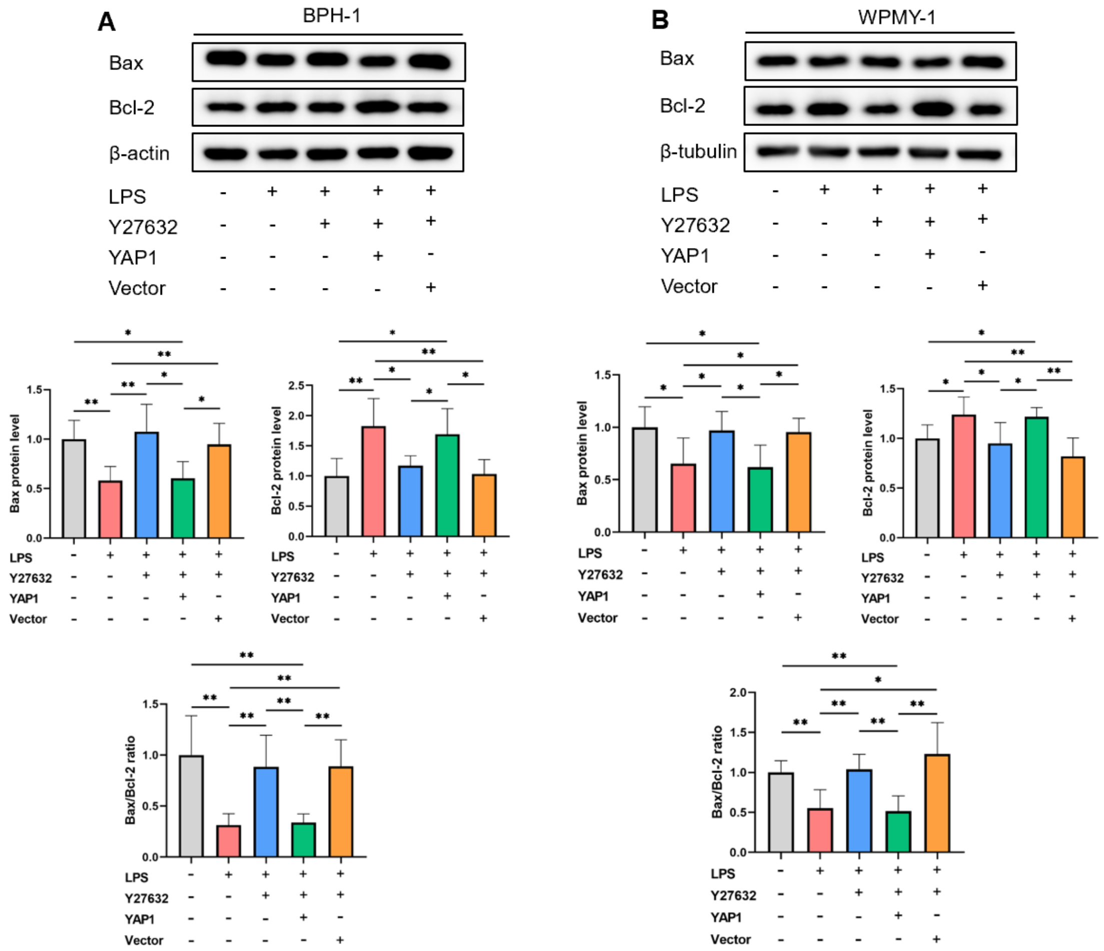

Figure S7. Involvement of ROCK1/YAP1 pathway in regulating cell apoptosis process. A, B) Immunoblotting analysis was performed to investigate the occurrence of apoptosis following treatment with the ROCK1 inhibitor (Y27632), YAP1 overexpression vector (YAP1), and control vector (vector) in BPH-1 and WPMY-1 cells. Data were presented as mean ± SD of at least 5 independent experiments. Two-tailed Student's *t*-test was used for A, B). *$p < 0.05$, ** $p < 0.01$.

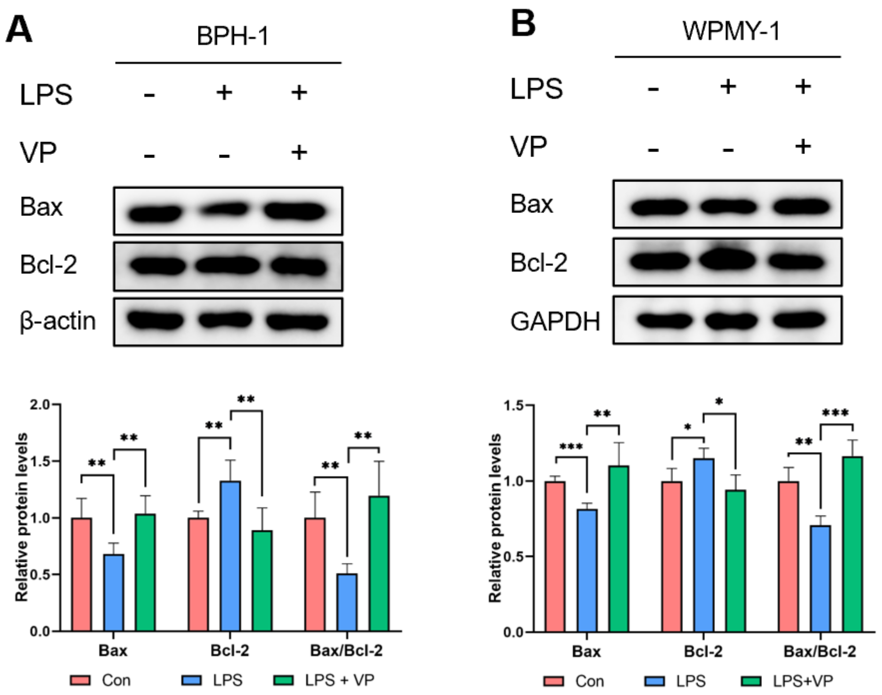

Figure S8. Blockade of YAP1-TEAD1 interaction triggers cell apoptosis in prostatic cells. A, B) Immunoblotting analysis to examine the curative effect of YAP1-TEAD1 binding inhibitor VP on apoptosis promotion. Data were presented as mean ± SD of at least 4 independent experiments. Two-tailed Student's *t*-test was used for A, B). $*p < 0.05$, $** p < 0.01$, $*** p < 0.001$.

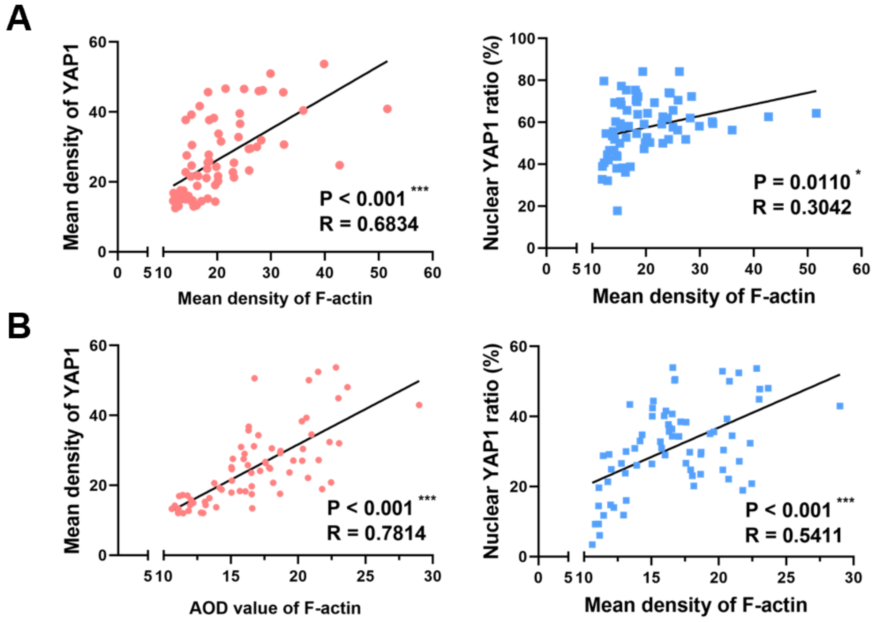

Figure S9. A positive association exists between actin polymerization and YAP1 intensity or nuclear YAP1 ratio. Correlation analysis to evaluate the association between F-actin intensity and YAP1 intensity or nuclear YAP1 ratio in both BPH-1 (A) and WPMY-1 (B) cells ($n \geq 24$ cells were recorded). Either Pearson or Spearmen correlation analysis was used for A, B). *$p < 0.05$, *** $p < 0.001$.
